# Effects of Dietary Digestible Energy Levels on Milk Performance, Rumen Fermentation, and Rumen Microbiota in Lactating Bactrian Camels from Southern Xinjiang

**DOI:** 10.3390/ani16193027

**Published:** 2026-09-25

**Authors:** Kui-Yuan Shao, Fei Han, Peng-Hui Chen, Hong-Bin Zhu, Bao-Rui Liu, Min Xu, Li-Lin Liu

**Affiliations:** 1 College of Animal Science and Technology, Tarim University, Alar 843300, China; 2 Key Laboratory of Livestock and Forage Resources Utilization Around Tarim, Ministry of Agriculture and Rural Affairs, Tarim University, Alar 843300, China; 3 Urumqi Animal Disease Control and Diagnosis Center, Urumqi 830063, China; 4 Urumqi Dairy Association, Urumqi 830063, China

**Keywords:** Bactrian camel, dietary digestible energy, lactation performance, rumen fermentation, rumen microbiota, metagenomic analysis

## Abstract

This study aimed to investigate the effects of different dietary digestible energy (DE) levels on lactation performance, rumen fermentation, and rumen microbiota in lactating Bactrian camels in southern Xinjiang. Twenty-four mid-lactation Bactrian camels were randomly assigned to four groups, with six camels per group, and were fed diets containing DE levels of 12.25 (CK), 12.49 (LE), 12.74 (ME), and 12.99 MJ/kg (HE), respectively. The experiment lasted 60 days, including a 15-day adaptation period and a 45-day experimental period. Metagenomic sequencing was performed to characterize the composition and functional gene profiles of the rumen microbiota. The results showed that increasing dietary DE significantly increased milk yield, milk fat yield, and lactose yield (*p* < 0.05), while decreasing ruminal NH_3_-N concentration. Propionate and butyrate concentrations were significantly higher in the HE group (*p* < 0.05). No significant differences were detected in rumen pH or the concentrations of acetate, valerate, and total volatile fatty acids among treatments (*p* > 0.05). No significant differences were detected in rumen microbial α-diversity or overall community structure among dietary DE treatments, although several bacterial genera showed differential enrichment. No significant differences were detected in the relative abundances of genes assigned to the major KEGG pathways or CAZy families among dietary treatments. In conclusion, within the dietary range examined in this study, higher DE was associated with greater lactation performance and changes in rumen fermentation, while no significant differences were detected in the overall rumen microbial community structure or the relative abundances of genes assigned to the major KEGG pathways and CAZy families.

## Simple Summary

This study evaluated the effects of dietary digestible energy levels of 12.25, 12.49, 12.74, and 12.99 MJ/kg on lactation performance, rumen fermentation, and rumen microbiota in lactating Bactrian camels. The results showed significant increases in milk yield, milk fat yield, and lactose yield with increasing dietary digestible energy, whereas no significant differences were detected in milk protein percentage, milk fat percentage, lactose percentage, or milk protein yield among treatments. As dietary digestible energy increased, ruminal ammonia nitrogen concentration decreased significantly, while propionate and butyrate concentrations were significantly higher in the high-energy group. In contrast, no significant differences were detected in acetate concentration, total volatile fatty acid concentration, or rumen pH among treatments. No significant differences were detected in overall rumen microbial diversity or community structure among dietary digestible energy treatments. Overall, within the dietary range examined in this study, an appropriate increase in digestible energy improved lactation performance and rumen fermentation in lactating Bactrian camels, while no significant differences were detected in overall rumen microbial diversity or community structure.

## 1. Introduction

Bactrian camels are important livestock species in the desert and semi-desert regions of Xinjiang, China, owing to their tolerance to poor-quality forage, drought resistance, and strong environmental adaptability, which enable them to survive in areas unsuitable for other livestock and to provide valuable sources of meat and milk [1]. Camel milk is considered a nutritious alternative for individuals with lactose intolerance and a functional food due to its rich content of bioactive compounds [2,3]. With the development of the camel milk industry, increasing attention has been paid to nutritional regulation and the improvement of lactation performance in lactating Bactrian camels.

Increasing dietary energy supply or concentrate supplementation can increase milk yield in lactating dromedary camels [4,5], although some studies have shown that increased energy intake does not necessarily result in a corresponding increase in milk production [6]. As the proportion of concentrate and dietary energy density increase, forage intake in lactating camels may decrease [7]. Under grazing conditions, increasing the level of concentrate supplementation has also been reported to markedly increase milk yield [8]. However, existing studies have focused primarily on concentrate supplementation and the regulation of energy intake in dromedary camels, whereas information on the effects of different dietary energy levels in lactating Bactrian camels remains limited.

Concentrate supplementation during the dry season has been reported to improve milk yield and milk quality in dromedary camels [9]. However, diets with excessively high energy density may promote body weight gain while reducing milk yield [10]. Although both species are foregut-fermenting camelids, differences in ecological adaptation and production environments mean that findings from dromedaries should be used as a reference rather than directly extrapolated to Bactrian camels. An appropriate dietary energy level can meet the requirements for maintenance, growth, and lactation in lactating animals, whereas insufficient or excessive energy supply may adversely affect productive performance and rumen fermentation [11,12,13]. Although metabolizable energy and net energy more directly reflect the energy available for metabolism and lactation, their calculation requires information on urinary and gaseous energy losses, and the estimation of net energy additionally requires parameters describing the efficiency of metabolizable energy utilization for maintenance and lactation. These parameters have not yet been adequately established for lactating Bactrian camels. Therefore, digestible energy, which relies on fewer species-specific parameters during energy conversion, was selected as the dietary energy index for comparing the different dietary treatments in the present study.

Dietary energy supply is closely associated with rumen fermentation because changes in fermentable substrate availability can alter microbial fermentation and volatile fatty acid production. Volatile fatty acids, mainly acetate, propionate, and butyrate, are the major end products of ruminal carbohydrate fermentation and represent an important source of energy for the host [14,15]. Changes in dietary energy density and forage-to-concentrate ratio can also modify the ruminal environment and substrate availability, thereby affecting microbial community composition and fermentation patterns [16,17,18]. Therefore, evaluating rumen fermentation and microbial communities is important for understanding the responses of lactating Bactrian camels to different dietary energy levels.

Although the effects of dietary energy levels on productive performance, rumen fermentation, and microbial communities have been widely investigated in dairy cows and small ruminants, related information on lactating Bactrian camels, especially those raised in Southern Xinjiang, remains scarce. From a practical production perspective, rational regulation of dietary digestible energy levels may help improve lactation performance and thereby enhance economic returns. We hypothesized that increasing dietary digestible energy would improve lactation performance and modulate rumen fermentation and microbial communities in lactating Bactrian camels. Therefore, this study was conducted to investigate the effects of different dietary digestible energy levels on milk production performance, rumen fermentation parameters, and rumen microbial communities in lactating Bactrian camels from Southern Xinjiang, with the aim of providing a theoretical basis for the rational regulation of dietary energy supply and improving feeding management strategies for lactating Bactrian camels.

## 2. Materials and Methods

### 2.1. Ethics Committee Approval

The experiment was conducted from July to September 2025 at Keping County Cooperative in Southern Xinjiang, China. The animal study was reviewed and approved by the Science and Technology Ethics Committee of Tarim University. The feeding process adhered to the “Ethical Review Guidelines for Laboratory Animal Welfare” (CHN).

### 2.2. Experimental Design

Twenty-four mid-lactation Bactrian camels were randomly assigned to four groups of six animals. Baseline milk yield did not differ significantly among groups, as shown in Supplementary Table S1. The control group (CK) was fed a diet with a digestible energy (DE) level of 12.25 MJ/kg, the low-energy group (LE) was fed a diet with a DE level of 12.49 MJ/kg, the medium-energy group (ME) was fed a diet with a DE level of 12.74 MJ/kg, and the high-energy group (HE) was fed a diet with a DE level of 12.99 MJ/kg. CK represented the basal control diet, whereas LE, ME, and HE represented progressively increased DE levels, with LE denoting the lowest DE level among the three energy-increased treatments. A relatively narrow DE interval of approximately 0.25 MJ/kg was used to establish a progressively increasing energy gradient while maintaining similar crude protein levels and avoiding large changes in overall dietary composition. The entire experimental period lasted 60 days, including a 15-day adaptation period and a 45-day experimental period. Before the experiment, the camel houses were dewormed and disinfected. The housing environments were similar among groups, and all camels were managed under a semi-confined feeding system. The experimental camels were group-fed twice daily at 09:00 and 19:00. Because group feeding was used in the present study, individual dry matter intake and nutrient intake could not be determined. Feed refusals were maintained at approximately 10% in each group, and residual feed was removed before each feeding. Milking was performed twice daily at 07:30 and 17:00, and calves were allowed to suckle for 1 min before milking.

### 2.3. Experimental Diets

The recommended metabolizable energy level for lactating dromedary camels is 9.00 MJ/kg, corresponding to a digestible energy level of 9.37 MJ/kg [19]. Bactrian camels are mainly distributed in cold regions between 35° and 48° N, whereas dromedary camels are distributed in hot regions ranging from 33° N to 40° S [20]. Previous physiological and genomic studies have revealed enhanced metabolic adaptations and thermogenesis-related genetic signals in Bactrian camels under cold conditions [21,22], suggesting greater energy expenditure for thermoregulation in cold environments. However, quantitative dietary energy requirements for lactating Bactrian camels have not yet been established. Therefore, recommendations for dromedary camels were used only as a general reference, and four graded DE levels were evaluated in the present study to characterize the responses of lactating Bactrian camels to increasing dietary energy supply. The ingredient and nutrient composition of the experimental diets are presented in Table 1.

### 2.4. Sample Collection and Measurements

#### 2.4.1. Milk Yield and Milk Composition

In adult female Bactrian camels, the gland cistern is highly underdeveloped and can store only approximately 100 mL of milk; therefore, suckling by the calf is required to stimulate milk let-down before milking. After the calves were allowed to suckle for 1 min, camel milk was collected using a vacuum milking machine (XD025 vacuum pump milker; Zibo Leniu Milking Equipment Co., Ltd., Zibo, China). The amount of milk consumed by the calves was not included in the recorded milk yield.

Although this may have slightly underestimated total milk production, the same standardized suckling procedure was applied to all animals and was therefore expected to have little effect on comparisons among treatment groups.

During the experimental period, camels were milked twice daily, and daily milk yield was calculated as the sum of the two milkings. Milk composition was measured every 3 days. The two milk samples collected each day were mixed at a ratio of 1:1, and the concentrations of milk fat, milk protein, lactose, and solids-not-fat in camel milk were determined using an milk composition analyzer (LM2-P1; Milkotester Ltd., Belovo, Bulgaria).

#### 2.4.2. Rumen Fluid Collection

Before morning feeding on day 60 of the experiment, rumen fluid was collected from all experimental Bactrian camels using the same standardized procedure with a rumen fluid collector. The first tube of rumen fluid was discarded, and the subsequent rumen fluid was collected for analysis. The rumen fluid was filtered through four layers of sterile gauze, and the clear filtrate was transferred into 10 mL centrifuge tubes. The pH was measured immediately using a portable pH meter (PHBJ-261L, Leici, Shanghai INESA Scientific Instrument Co., Ltd., Shanghai, China). The rumen fluid was then divided into two portions. One portion was placed in 2.5 mL centrifuge tubes and stored at −20 °C for the determination of volatile fatty acids (VFAs) and ammonia nitrogen (NH_3_-N) concentrations. The other portion was snap-frozen in liquid nitrogen and rapidly transferred to a −80 °C freezer for shotgun metagenomic sequencing.

#### 2.4.3. Analysis of Rumen Fermentation Parameters

Owing to the persistent solid–liquid stratification of camel rumen contents, only a limited volume of rumen fluid could be collected. Therefore, twofold-diluted rumen fluid was used for the determination of volatile fatty acids (VFAs) and ammonia nitrogen (NH_3_-N). The dilution factor was accounted for when calculating the final concentrations, and all reported VFA and NH3-N values represent concentrations in the original, undiluted rumen fluid.

The pretreatment of rumen fluid for VFA analysis was performed as follows: Briefly, 10 mL of diluted rumen fluid was centrifuged at 12,000 r/min for 15 min. After centrifugation, 1.5 mL of the supernatant was collected, mixed with 0.15 mL of 25% metaphosphoric acid at a ratio of 10:1, and transferred into a 2 mL centrifuge tube. The mixture was then centrifuged at 15,000 r/min for 10 min, and the supernatant was collected. The supernatant was passed through a 0.22 μm aqueous-phase membrane filter using a syringe, and the filtrate was transferred into amber glass vials for subsequent analysis. VFAs were determined according to the method of Erwin et al. [24] using an Agilent 6890N gas chromatograph (Agilent Technologies, Santa Clara, CA, USA). The concentration of NH_3_-N was determined using the phenol–sodium hypochlorite colorimetric method described by Feng Zongci [25].

### 2.5. Rumen Metagenomic Sequencing and Bioinformatics Analysis

#### 2.5.1. DNA Extraction, Library Construction, and Sequencing

Rumen metagenomic sequencing was performed by Tsingke Biotechnology Co., Ltd. (Beijing, China). Total DNA was extracted from the samples using the PowerSoil^®^ DNA Isolation Kit and quantified using a Qubit fluorometer (Invitrogen, Carlsbad, CA, USA). Qualified DNA was randomly fragmented to approximately 300–350 bp, followed by end repair, A-tailing, adapter ligation, and PCR amplification to construct sequencing libraries using the TrueLib DNA Library Rapid Prep Kit for Illumina (ExCell Bio, Shanghai, China). Library quality was assessed using an Agilent 2100 Bioanalyzer (Agilent Technologies, Santa Clara, CA, USA) and quantified using a Qubit 3.0 Fluorometer (Life Technologies, Carlsbad, CA, USA). After library quality control, the samples were sequenced on an Illumina NovaSeq 6000 platform (Illumina, San Diego, CA, USA) using a paired-end 150-bp sequencing strategy.

#### 2.5.2. Sequencing Data Quality Control

Raw sequencing reads were initially evaluated using FastQC (v0.12.1), and adapter trimming and quality filtering of the paired-end reads were performed using fastp (v1.0.1). Reference genomes of Bactrian camel (Camelus bactrianus, GCF_048773025.1) and major dietary plant ingredients, including maize (Zea mays, GCF_902167145.1), alfalfa (Medicago sativa, GCF_051527215.1), and soybean (Glycine max, GCF_000004515.6), were downloaded from the NCBI database. Genome indexes were constructed using Bowtie2 (v2.2.4), and reads mapped to the Bactrian camel genome or feed-derived plant genomes were removed to minimize contamination from host and dietary DNA. The remaining unmapped reads were retained for downstream metagenomic analyses.

#### 2.5.3. Taxonomic Annotation and Diversity Analysis

Taxonomic annotation was performed using Kraken2 based on the GTDB v226 database. Bracken was then used to re-estimate taxonomic abundance by applying a Bayesian algorithm to redistribute the classification results generated by Kraken2, thereby obtaining a more accurate relative abundance profile of microbial communities. Alpha diversity indices were calculated using the vegan package (v2.7.5) in R software (v4.5.2). Principal coordinate analysis (PCoA) based on the Bray–Curtis distance matrix was performed to evaluate differences in beta diversity among samples. Differentially abundant taxa among dietary treatments were identified using linear discriminant analysis effect size (LEfSe), with *p* < 0.05 and an LDA score > 2.0 considered significant.

#### 2.5.4. Functional Annotation and Metabolic Pathway Analysis

Metagenomic reads were assembled using metaSPAdes (v4.2.0), and protein-coding genes were predicted using Prodigal (v2.6.3) in metagenomic mode. Functional annotation based on the KEGG Orthology system was performed using KofamScan (v1.3.0). Carbohydrate-active enzyme genes were annotated using dbCAN (v3.0.7) against the CAZy database. Gene abundances were normalized as TPM.

### 2.6. Statistical Analysis

The experimental data were initially organized using Microsoft Excel 2019. One-way analysis of variance (ANOVA) was performed using SPSS 22.0, and differences among groups were compared using Duncan’s multiple range test. Because dietary digestible energy levels were arranged as graded treatments, linear and quadratic trend analyses were performed to evaluate the responses of measured variables to increasing dietary digestible energy levels. Individual camels served as observational units for animal-level measurements, with six camels per treatment. However, the six camels within each treatment were group-fed in a single feeding unit; therefore, dietary treatments were not independently replicated at the feeding-unit level. For milk yield and milk composition, repeated measurements collected during the experimental period were averaged for each camel before statistical analysis. Alpha-diversity indices were compared using one-way ANOVA, and beta-diversity was assessed using ANOSIM based on Bray–Curtis dissimilarity. LEfSe was used to identify differential taxa, and genus-level relative abundance comparisons were performed using the Kruskal–Wallis test, with *p*-values adjusted using the Benjamini–Hochberg false discovery rate procedure, while KEGG pathways and CAZy families were analyzed using one-way ANOVA, with Dunnett’s test used for CAZy comparisons against the CK group. The results are expressed as means with the standard error of the mean (SEM). Figures were generated using R software (v4.5.2) and related packages. Differences were considered significant at *p* < 0.05 and highly significant at *p* < 0.01.

## 3. Results

### 3.1. Effects of Dietary Digestible Energy Levels on Milk Production Performance

As shown in Table 2, milk yield was significantly lower in the CK group than in the LE, ME, and HE groups (*p* < 0.01), and a significant positive linear effect of dietary digestible energy level on milk yield was observed (*p* < 0.01). Milk fat yield was significantly lower in the CK group than in the ME and HE groups (*p* < 0.05) and showed a significant positive linear response to increasing dietary digestible energy levels (*p* < 0.01). Lactose yield was significantly lower in the CK group than in the LE, ME, and HE groups (*p* < 0.01) and was also significantly lower in the LE group than in the ME and HE groups (*p* < 0.01). A significant positive linear effect of dietary digestible energy level on lactose yield was observed (*p* < 0.01). No significant differences were observed among the groups in milk protein percentage, milk protein yield, milk fat percentage, lactose percentage, or solids-not-fat content (*p* > 0.05).

### 3.2. Effects of Dietary Digestible Energy Levels on Rumen Fermentation Parameters

As shown in Table 3, ruminal pH, acetate concentration, valerate concentration, the acetate-to-propionate ratio, and total volatile fatty acid concentration did not differ significantly among the dietary treatments (*p* > 0.05). Ruminal NH_3_-N concentration was lower in the HE group than in the CK and LE groups (*p* < 0.01), while that in the ME group was lower than in the CK group (*p* < 0.01). At the *p* < 0.05 level, both the ME and HE groups had lower NH_3_-N concentrations than the CK and LE groups. Propionate concentration was higher in the HE group than in the CK and LE groups (*p* < 0.05), whereas butyrate concentration was higher in the HE group than in the CK, LE, and ME groups (*p* < 0.05). As dietary digestible energy level increased, NH_3_-N concentration decreased linearly (*p* < 0.01), whereas propionate and butyrate concentrations increased linearly (*p* < 0.01). The acetate-to-propionate ratio also exhibited a significant linear response to increasing dietary digestible energy levels (*p* < 0.01).

### 3.3. Effects of Dietary Digestible Energy Levels on Rumen Microbial Diversity

As shown in Figure 1A, the rarefaction curves for all samples approached a plateau, indicating that the sequencing depth was sufficient to characterize the rumen microbial communities. No significant differences were detected in the α-diversity indices of the rumen microbiota, including observed genera, Shannon index, and Simpson index, among dietary digestible energy treatments (*p* > 0.05; Figure 1B–D). In the principal coordinate analysis, the first and second principal coordinates explained 42.00% and 21.83% of the total variation, respectively, and no distinct separation was observed among the four groups (Figure 1E). Consistently, ANOSIM yielded an R value of −0.048 and revealed no significant differences in community dissimilarity among the groups (*p* > 0.05; Figure 1F); therefore, no significant differences in the overall rumen microbial community structure were detected among dietary digestible energy treatments.

At the phylum level, the rumen microbial community was predominantly composed of Bacteroidota and Bacillota, followed by Pseudomonadota, Spirochaetota, and other less abundant phyla, with broadly similar community compositions among the groups (Figure 1G). At the genus level, *Prevotella* was the predominant genus, whereas the other dominant genera occurred at relatively lower abundances. The relative abundance profiles of the major genera were generally similar among the four groups (Figure 1H).

LEfSe analysis revealed five bacterial genera exhibiting differential enrichment patterns among dietary digestible energy levels, as shown in Figure 2. Specifically, *Eubacterium_G* and *Spyradosoma* were characteristic taxa enriched in the CK group, *UBA3206* was enriched in the LE group, and *UMGS1933* and *Brevundimonas* were enriched in the HE group. Subsequent relative abundance analysis showed that, after Benjamini–Hochberg false discovery rate correction for multiple testing, none of the five genera differed significantly among dietary treatments (adjusted *p* > 0.05). No significant pairwise differences were detected after correction.

### 3.4. Effects of Dietary Digestible Energy Levels on Rumen Microbial Metabolic Pathways of Lactating Bactrian Camels in Southern Xinjiang

As shown in Figure 3A, no significant differences were detected in the relative abundances of genes assigned to the selected KEGG pathways among the dietary groups. The 30 representative CAZy gene families exhibited varying abundance patterns across dietary digestible energy levels; however, none of these differences reached statistical significance (*p* > 0.05). No significant differences were detected in the relative abundances of genes assigned to the major ruminal microbial CAZy families among dietary digestible energy treatments.

## 4. Discussion

### 4.1. Effects of Dietary Digestible Energy Levels on Lactation Performance of Bactrian Camels in Southern Xinjiang

Forage nutrient quality, water availability, climatic conditions, milking management, and animal health status can all affect milk production performance in camels [26,27]. The average daily milk yield of dromedary camels is approximately 6 kg, with peak yields reaching 8.9 kg/d [28], whereas the average daily milk yield of Bactrian camels in China is approximately 1.0–2.0 kg/d [29]. In the present study, the daily milk yield of Bactrian camels in southern Xinjiang ranged from 2.79 to 3.24 kg, and such differences may be mainly attributable to genetic background and lactation potential, as well as differences in diet, management, lactation stage, and milking practices. Increasing dietary energy supply generally favors milk production in lactating animals. Previous research showed that increasing the total digestible nutrient content of diets for lactating camels from 50% to 60% increased milk yield [30]. Faraz et al. [26] reported that concentrate supplementation alone did not significantly affect milk fat, protein, lactose, or solids-not-fat contents in dromedary camels, whereas milk fat percentage increased significantly when concentrate supplementation was combined with microbial metabolite additives. Moreover, additional concentrate supplementation has been reported to significantly increase milk yield in dromedary camels [14]. These findings indicate that increasing dietary energy concentration or concentrate supply contributes to increased milk yield. In the present study, milk yield in Bactrian camels increased significantly with increasing dietary DE concentration; however, because individual feed intake was not measured, it was not possible to distinguish whether the observed differences were attributable to dietary energy density, voluntary feed intake, or total energy intake.

Lactose accounts for approximately 53.8% of the total osmotic pressure of milk and is the principal osmotic component responsible for maintaining milk osmolarity [31]. In comparison, milk protein makes only a minor direct contribution to osmotic pressure, whereas the direct osmotic effect of milk fat is negligible [32]. Increased lactose synthesis promotes the osmotic movement of water into the mammary lumen, and mammary lactose is therefore a key determinant of milk synthesis and secretion [33]. In the present study, no significant differences in lactose percentage were detected among dietary digestible energy treatments, whereas lactose yield increased significantly. This pattern may be associated with greater lactose synthesis accompanying the increase in milk yield, although lactose synthesis was not directly measured. Milk fat percentage did not differ significantly among treatments, whereas milk fat yield increased concomitantly with milk yield, which may partly reflect a dilution effect associated with greater milk volume. Beyond increasing milk yield, nutritional strategies may also contribute to improving camel milk quality [9,34]. Meanwhile, emerging spectroscopic techniques provide rapid approaches for assessing the quality and authenticity of camel milk products [35,36].

### 4.2. Effects of Dietary Digestible Energy Levels on Rumen Fermentation Parameters of Lactating Bactrian Camels in Southern Xinjiang

Rumen pH is jointly regulated by dietary substrate fermentation and microbial metabolism. Volatile fatty acids (VFAs) are the major end products of ruminal carbohydrate fermentation, and their production and accumulation are generally accompanied by a decrease in rumen pH [33,37,38]. In the present study, rumen pH decreased numerically with increasing dietary energy level, but no significant differences were detected among treatments. Similarly, no significant differences in total VFA concentration were detected among dietary treatments under the conditions of this study.

Dietary energy level can influence ruminal NH_3_-N concentration by altering the energy supply available to rumen microorganisms and their efficiency of nitrogen utilization [39,40]. Bekele et al. [41] reported that ruminal NH_3_-N concentration in dromedary camels initially increased and then decreased as the proportion of dietary concentrate increased. In the present study, ruminal NH_3_-N concentration generally decreased with increasing dietary energy level. One possible explanation is more efficient microbial utilization of rumen-available nitrogen; however, microbial protein synthesis was not directly measured in this study, and this mechanism therefore remains hypothetical and requires further verification.

Feeds rich in structural carbohydrates, such as cellulose and lignin, are degraded relatively slowly in the rumen and tend to favor acetate production, whereas non-structural carbohydrates, such as starch and simple sugars, are rapidly fermented and generally favor propionate production [42,43]. Agle et al. [17] found that increasing the proportion of concentrate in the diets of lactating dairy cows reduced rumen pH and ammonia concentration, increased propionate concentration, and decreased the acetate-to-propionate ratio. Liu et al. [44] also reported that increasing dietary concentrate significantly reduced the proportion of acetate in the rumen.

In the present study, increasing dietary DE was accompanied by increased starch content and decreased NDF content. Similar changes in dietary carbohydrate composition have been reported to favor propionate production and decrease the acetate-to-propionate ratio [45,46]. Therefore, the observed fermentation responses may reflect both the increase in dietary energy concentration and the associated changes in diet composition. In the present study, as the dietary energy level increased, acetate concentration decreased numerically, whereas propionate concentration increased, resulting in a corresponding numerical decline in the acetate-to-propionate ratio.

### 4.3. Effects of Dietary Digestible Energy Levels on Rumen Microbial Diversity of Lactating Bactrian Camels in Southern Xinjiang

The rarefaction curves of species richness in all groups approached a plateau, indicating that the sequencing depth was sufficient to adequately characterize the rumen microbial communities. No significant differences were detected in the number of observed genera, Shannon index, or Simpson index among dietary energy treatments. Wu et al. [47] reported a similar finding in Small-Tailed Han sheep and Tibetan sheep fed diets containing four metabolizable energy levels of 6.73, 7.65, 8.57, and 9.49 MJ ME/kg DM, with no significant effects on α-diversity.

Principal coordinate analysis (PCoA) based on Bray–Curtis dissimilarity showed no clear separation among the treatment groups. Consistently, ANOSIM analysis detected no significant differences in rumen microbial community structure among dietary digestible energy treatments, consistent with the results of the α-diversity analysis.

Bacteroidota, Bacillota, Pseudomonadota, and Spirochaetota are the major bacterial phyla in the camel rumen, with Bacteroidota and Bacillota generally predominating [48]. Similarly, Bacteroidota and Bacillota are also the dominant bacterial phyla in the rumen microbiota of sheep [49], suggesting a certain degree of conservation in rumen microbial composition at the phylum level across different ruminant species. In the present study, Bacteroidota and Bacillota were likewise the predominant phyla in all treatment groups, consistent with previous findings. At the genus level, *Prevotella* was the dominant genus across all treatments. *Prevotella* has also been reported as a predominant rumen genus in sheep fed high-energy diets [49]. In goats, the relative abundance of *Prevotella* has been positively correlated with milk yield and milk fat percentage [50]. In the present study, milk yield increased with increasing dietary digestible energy level, whereas the relative abundance of *Prevotella* initially increased and subsequently decreased in the HE group. However, the present results do not establish a direct functional relationship between Prevotella abundance and milk production, and this association should therefore be interpreted cautiously.

*Eubacterium_G* is a genus delineated from the traditional *Eubacterium* group by the Genome Taxonomy Database (GTDB) based on genome-scale phylogeny. Previous studies in ruminants have linked *Eubacterium* with cellulose utilization, and members of this genus have been reported to participate in ruminal cellulose degradation [51,52]. However, the taxonomic abundance of *Eubacterium_G* observed in the present study does not by itself demonstrate a functional role in cellulose degradation, and further functional evidence is required to confirm this association.

### 4.4. Effects of Dietary Digestible Energy Levels on Rumen Microbial Metabolic Pathways and Carbohydrate-Active Enzyme Families of Lactating Bactrian Camels in Southern Xinjiang

The selected KEGG functional pathways were mainly associated with carbohydrate metabolism, energy metabolism, and environmental information processing. Among these pathways, glycolysis/gluconeogenesis, starch and sucrose metabolism, pyruvate metabolism, propanoate metabolism, and butanoate metabolism are closely associated with ruminal carbohydrate fermentation and volatile fatty acid production [53,54]. No significant differences were detected in the relative abundances of genes assigned to these KEGG pathways among dietary digestible energy treatments. Metagenomic sequencing reflects functional gene potential rather than actual microbial activity; therefore, these results should not be interpreted as evidence that metabolic activity remained unchanged. Similarly, no significant differences were detected in the relative abundances of genes assigned to representative CAZy families, including GH, GT, CE, CBM, PL, and AA, among treatments. Therefore, within the dietary energy range examined in this study, no significant differences were detected in the relative abundance profiles of major carbohydrate-active enzyme-related genes.

## 5. Conclusions

Increasing the dietary digestible energy concentration from 12.25 to 12.99 MJ/kg was associated with increased milk yield and changes in rumen fermentation in lactating Bactrian camels, as evidenced by increased propionate and butyrate concentrations and decreased ruminal NH_3_-N concentration. However, no significant differences were detected in overall rumen microbial diversity, community structure, or the relative abundances of genes assigned to selected KEGG pathways or CAZy families. These findings indicate that higher dietary digestible energy concentrations within the range examined were associated with greater lactation performance, while no significant differences were detected in the overall rumen microbial community structure or the relative abundances of genes assigned to the selected KEGG pathways and CAZy families.

## Figures and Tables

**Figure 1:**
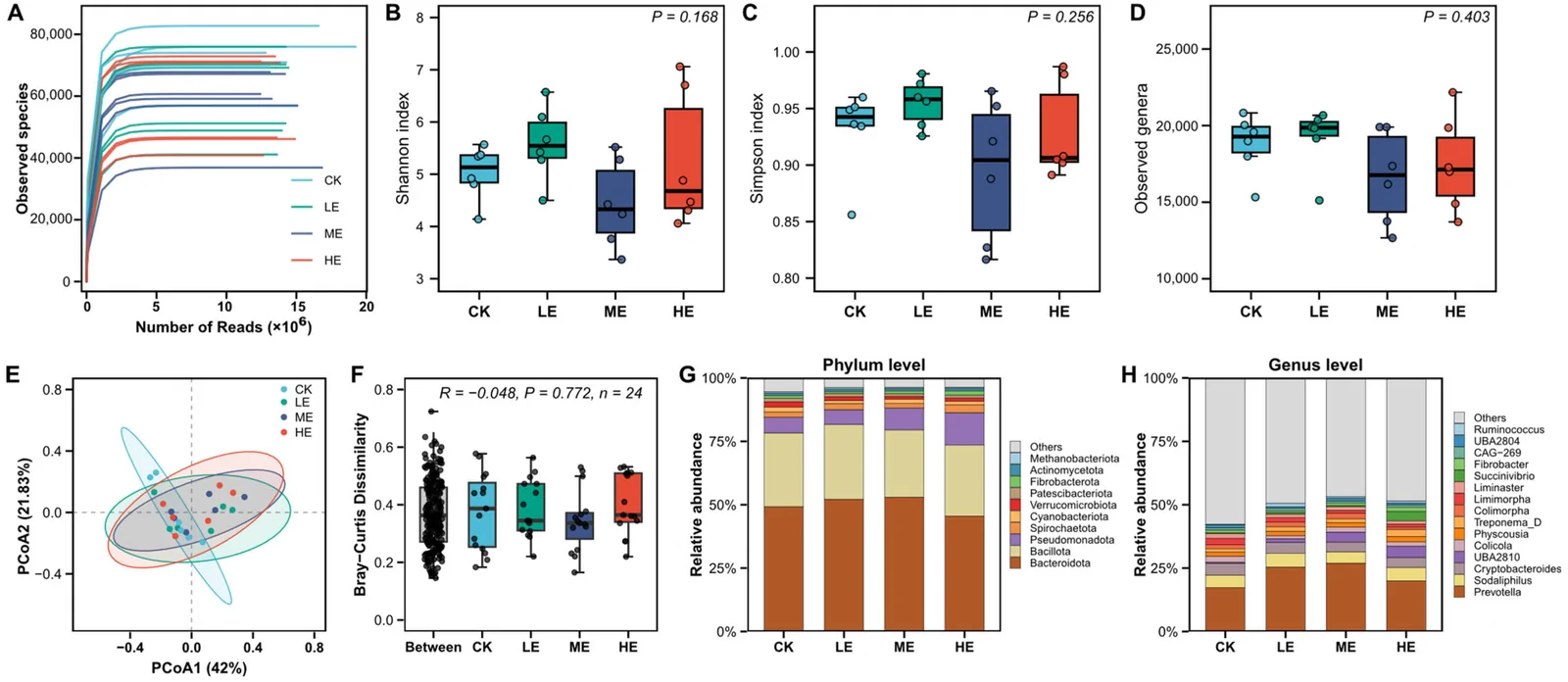
Effects of dietary digestible energy levels on rumen microbial diversity and community composition in lactating Bactrian camels: (**A**) rarefaction curves of rumen microbial communities; (**B**–**D**) alpha-diversity indices, including the Shannon index, Simpson index, and observed genera, respectively; (**E**) principal coordinate analysis based on Bray–Curtis dissimilarities; (**F**) analysis of similarities based on Bray–Curtis dissimilarities; (**G**,**H**) relative abundances of the major rumen microbial taxa at the phylum and genus levels, respectively.

**Figure 2:**
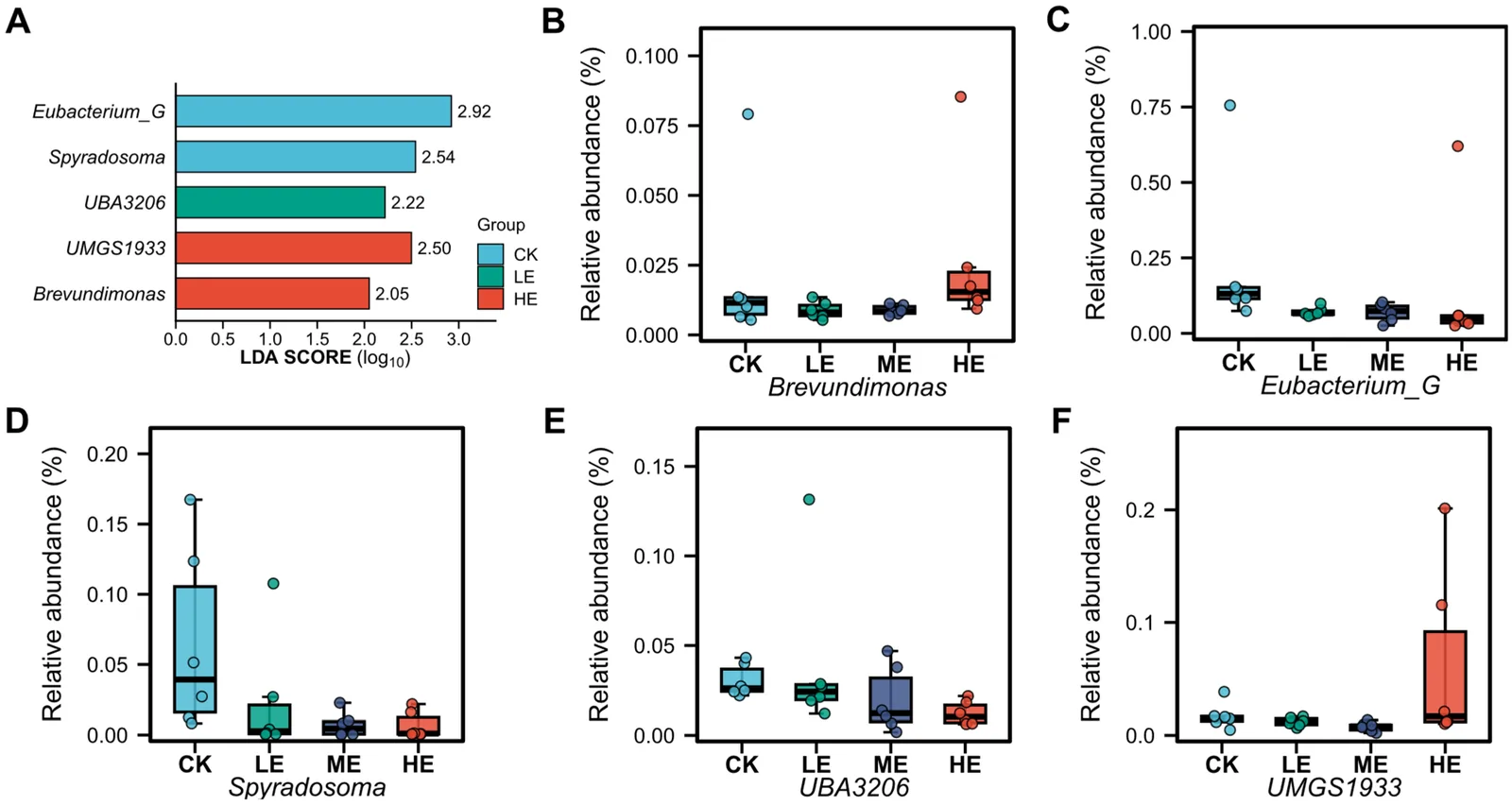
Effects of dietary digestible energy levels on LEfSe-identified differential bacterial genera and their relative abundance distributions in the rumen of lactating Bactrian camels: (**A**) Differential bacterial genera identified by LEfSe analysis and their corresponding linear discriminant analysis (LDA) scores. (**B**–**F**) Relative abundance distributions of the differential genera among the four groups, including *Brevundimonas* (**B**), *Eubacterium_G* (**C**), *Spyradosoma* (**D**), *UBA3206* (**E**), and *UMGS1933* (**F**).

**Figure 3:**
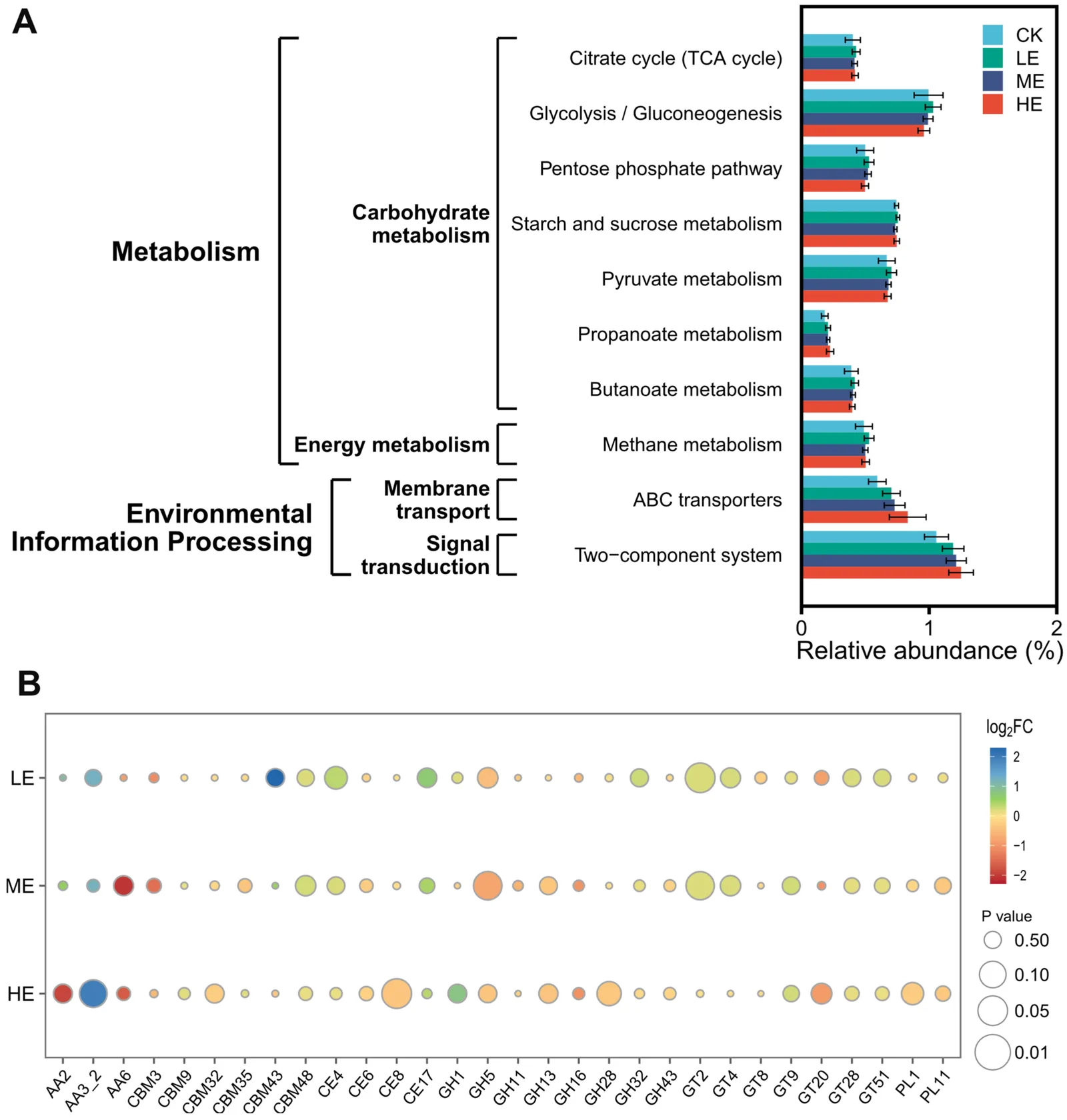
Effects of dietary digestible energy levels on the relative abundances of genes assigned to selected ruminal microbial KEGG pathways and CAZy families in lactating Bactrian camels: (**A**) Relative abundances of genes assigned to selected KEGG Level 3 pathways. (**B**) Bubble plot showing differences in 30 representative CAZy gene families in the LE, ME, and HE groups relative to the CK group. Bubble color represents the log_2_ fold change in abundance relative to the CK group, whereas bubble size represents the *p*-value obtained from Dunnett’s test.

**Table 1:** Feed ingredient and nutrient composition (dry matter basis, DM%).

Items	CK	LE	ME	HE
Ingredients				
Rapeseed meal	1.80	1.80	1.62	0.90
Wheat bran	2.30	2.30	2.07	1.15
Corn silage	59.32	58.33	57.35	56.38
Soybean meal	6.45	6.45	7.29	8.67
Alfalfa	21.56	16.81	11.87	9.90
Corn	6.97	11.92	16.46	20.81
DDGS	0.40	0.40	0.36	0.20
Wheat straw	0.20	0.99	1.98	0.99
Premix ^(1)^	1.00	1.00	1.00	1.00
Total	100.00	100.00	100.00	100.00
Nutrient levels				
DM,% as-fed	40.94	41.29	41.65	42.01
CP	13.19	13.19	13.20	13.20
EE	2.48	2.61	2.70	2.69
Starch	21.28	24.41	27.21	29.64
ADF	28.88	27.19	25.44	23.66
NDF	47.11	44.97	42.86	40.53
Ash	6.40	5.98	5.58	5.31
DE,(MJ/kg) ^(2)^	12.25	12.49	12.74	12.99

^(1)^ The premix used in the experiment provided the following nutrients per kilogram of complete diet: vitamin A, 18,000 IU; vitamin D3, 3000 IU; vitamin E, 45 mg; iron, 200 mg; copper, 15 mg; zinc, 100 mg; manganese, 100 mg; selenium, 0.3 mg; iodine, 0.5 mg; and cobalt, 0.1 mg. ^(2)^ Because no validated DE prediction system is currently available for Bactrian camels, digestible energy was calculated for all experimental diets using the NRC (2001) dairy cow model [23], whereas the other values were measured.

**Table 2:** Effects of dietary digestible energy levels on lactation parameters.

Items	CK	LE	ME	HE	SEM	*p*-Value		
						Treatment	Linear	Quadratic
Milk yield (kg/d)	2.79 ^Bc^	3.05 ^Ab^	3.19 ^Aab^	3.24 ^Aa^	0.065	<0.001	<0.001	0.101
Milk protein (%)	4.01	3.95	3.84	3.85	0.159	0.849	0.415	0.828
Milk protein yield (g/d)	112.05	120.68	122.59	124.91	4.946	0.306	0.082	0.530
Milk fat (%)	4.21	4.17	4.16	4.07	0.119	0.862	0.482	0.836
Milk fat yield (g/d)	117.53 ^b^	127.24 ^ab^	133.06 ^a^	131.96 ^a^	3.736	0.032	0.008	0.163
Lactose (%)	5.66	5.60	5.68	5.76	0.062	0.354	0.183	0.271
Lactose yield (g/d)	157.99 ^C^	170.82 ^B^	181.35 ^A^	186.74 ^A^	1.925	<0.001	<0.001	0.068
Solids-not-fat (%)	11.07	11.05	11.21	11.12	0.128	0.815	0.591	0.790

Within the same row, different lowercase letters indicate significant differences (*p* < 0.05), and different uppercase letters indicate highly significant differences (*p* < 0.01).

**Table 3:** Effects of different digestible energy levels on rumen fermentation parameters.

Items	CK	LE	ME	HE	SEM	*p*-Value		
						Treatment	Linear	Quadratic
pH	7.03	6.99	6.74	6.67	0.139	0.512	0.183	0.967
NH_3_-N (mg/L)	152.63 ^aA^	143.56 ^aAB^	128.45 ^bBC^	118.48 ^bC^	4.561	<0.001	<0.001	0.922
Acetate (mmol/L)	59.22	58.71	57.30	55.84	1.298	0.281	0.061	0.171
Propionate (mmol/L)	14.03 ^b^	14.91 ^b^	16.10 ^ab^	17.37 ^a^	0.749	0.027	0.005	0.791
Butyrate (mmol/L)	6.25 ^b^	6.91 ^b^	6.71 ^b^	7.82 ^a^	0.302	0.012	0.003	0.471
Valerate (mmol/L)	1.01	1.10	1.07	1.18	0.065	0.341	0.116	0.868
A:P ratio	4.33	3.96	3.61	3.29	0.269	0.067	0.009	0.996
TVFAs (mmol/L)	80.50	81.61	81.17	82.19	0.886	0.597	0.257	0.962

Abbreviations: NH_3_-N, ammonia nitrogen; A:P ratio, acetate-to-propionate ratio; TVFAs, total volatile fatty acids. Within the same row, different lowercase letters indicate significant differences (*p* < 0.05), and different uppercase letters indicate highly significant differences (*p* < 0.01).

## Data Availability

The original contributions presented in this study are included in the article and its Supplementary Materials. The raw metagenomic sequencing data generated in this study have been deposited in the NCBI Sequence Read Archive (SRA) under BioProject accession number PRJNA1519050. Further inquiries can be directed to the corresponding author.

## Supplementary Materials

The following supporting information can be downloaded at https://www.mdpi.com/article/10.3390/ani16193027/s1, Figure S1: LEfSe analysis of differential rumen bacterial species in lactating Bactrian camels fed diets with different digestible energy levels; Figure S2: Circular phylogenetic tree of putatively novel MAGs from the camel rumen; Table S1. Baseline milk yield of lactating Bactrian camels in the four treatment groups; Table S2: Summary of raw and clean metagenomic sequencing data from rumen samples; Table S3: Relative abundances of the top 20 dominant rumen microbial phyla in lactating Bactrian camels fed diets with different digestible energy levels; Table S4: Relative abundances of the top 20 dominant rumen bacterial genera in lactating Bactrian camels fed diets with different digestible energy levels; Table S5. Genus-level *p*-values after FDR correction; Table S6: Detection of lignin-associated CAZymes in candidate MAGs.
