# Supplementary material for: Effects of Dietary Digestible Energy Levels on Milk Performance, Rumen Fermentation, and Rumen Microbiota in Lactating Bactrian Camels from Southern Xinjiang
Source: Animals (Basel). 2026 Sep 25;16(19):3027. doi: 10.3390/ani16193027 (PMC13663552; doi:10.3390/ani16193027)
Supplement: Supplementary file 1 [file animals-16-03027-s001.zip › Supplementary_Materials.pdf]

Table S1. Baseline milk yield of lactating Bactrian camels in the four treatment groups.

Items	CK	LE	ME	HE	*P*
Milk yield, kg/d	2.48±0.14	2.47±0.16	2.45±0.15	2.49±0.16	0.981

Values are presented as mean ± standard deviation

Table S2. Summary of Raw and Clean Metagenomic Sequencing Data from Rumen Samples

DisplayID	Raw reads(M)	Raw Q30 (%)	Raw GC (%)	Raw average length (bp)	Clean reads (M)	Clean Q30 (%)	Clean GC (%)	Clean average length (bp)	Clean rate (%)
CK1	60.08	92.88	45.39	150	56.91	95.54	44.98	147.0	94.73
CK2	44.2	90.52	44.88	150	40.43	95.06	44.05	144.0	91.45
CK3	46.87	90.68	47.95	150	42.77	95.00	47.22	144.5	91.25
CK4	54.99	91.66	34.18	150	51.12	95.90	32.74	142.5	92.98
CK5	46.02	90.50	32.96	150	42.10	95.49	31.22	142.5	91.48
CK6	51.22	91.67	43.49	150	47.99	94.79	42.98	147.0	93.68
LE1	47.14	92.37	44.35	150	44.12	95.41	43.85	147.5	93.59
LE2	48.56	90.69	42.92	150	44.50	94.60	42.15	147.0	91.65
LE3	45.11	90.76	45.76	150	41.34	95.19	45.04	144.5	91.64
LE4	46.08	90.97	46.66	150	42.34	95.21	46.06	145.0	91.89
LE5	46.83	90.64	43.01	150	42.90	94.87	42.23	145.5	91.6
LE6	48.46	91.43	36.08	150	44.27	95.62	34.67	145.0	91.36
ME1	44.93	90.98	39.74	150	41.12	94.90	38.79	146.5	91.51
ME2	46.89	91.60	41.44	150	43.76	95.28	40.70	145.0	93.32
ME3	49.79	91.92	44.29	150	45.97	95.53	43.60	146.0	92.33
ME4	43.51	91.06	40.11	150	40.13	95.30	39.19	145.0	92.24
ME5	44.30	92.52	44.48	150	41.77	95.32	44.04	147.0	94.29
ME6	52.05	91.52	47.96	150	47.65	95.39	47.17	146.0	91.55
HE1	45.5	90.98	41.30	150	42.13	95.20	40.43	144.0	92.6.0
HE2	43.77	90.94	39.13	150	40.10	95.46	37.95	143.0	91.62
HE3	44.87	90.99	45.54	150	41.33	94.87	44.97	146.0	92.13
HE4	46.61	91.54	45.18	150	43.01	95.58	44.49	145.0	92.28
HE5	42.11	91.39	43.68	150	39.17	95.09	43.10	145.5	93.0
HE6	52.62	91.26	44.95	150	48.67	95.13	44.37	145.5	92.48

Table S3. Relative Abundances of the Top 20 Dominant Rumen Microbial Phyla in Lactating Bactrian Camels Fed Diets with Different Digestible Energy Levels

Taxa	CK	LE	ME	HE	*P*
Bacteroidota	49.14	52.17	52.95	45.53	0.465
Bacillota	29.21	29.48	26.58	28.00	0.607
Pseudomonadota	6.18	5.87	8.62	12.80	0.379
Spirochaetota	2.09	2.33	1.89	3.14	0.343
Cyanobacteriota	1.87	1.35	1.58	1.38	0.722
Verrucomicrobiota	2.02	1.31	1.07	1.24	0.169
Patescibacteriota	1.53	1.35	1.12	1.14	0.425
Fibrobacterota	1.00	0.81	1.14	1.71	0.223
Actinomycetota	1.03	0.94	0.91	0.95	0.643
Methanobacteriota	0.56	0.59	0.47	0.45	0.718
Campylobacterota	0.52	0.33	0.33	0.32	0.677
Bacteroidota_A	0.49	0.31	0.31	0.30	0.656
Planctomycetota	0.30	0.24	0.23	0.26	0.320
Chloroflexota	0.29	0.23	0.22	0.24	0.363
Elusimicrobiota	0.32	0.24	0.23	0.14	0.173
Acidobacteriota	0.26	0.22	0.21	0.23	0.280
Desulfobacterota	0.28	0.20	0.20	0.20	0.603
Thermoplasmatota	0.20	0.15	0.13	0.17	0.794
Nanobdellota	0.22	0.14	0.14	0.14	0.704
Thermoproteota	0.20	0.13	0.13	0.13	0.698

Table S4. Relative Abundances of the Top 20 Dominant Rumen Bacterial Genera in Lactating Bactrian Camels Fed Diets with Different Digestible Energy Levels

Taxa	CK	LE	ME	HE	*P*
Prevotella	17.32	25.44	26.97	19.99	0.169
Sodaliphilus	4.83	5.39	4.41	5.24	0.967
Cryptobacteroides	4.79	4.27	3.92	4.03	0.921
UBA2810	0.31	1.45	3.81	4.35	0.340
Colicola	2.49	1.31	2.05	1.76	0.341
Physcousia	1.59	1.71	1.84	2.14	0.715
Treponema_D	1.34	1.73	1.44	2.70	0.293
Colimorpha	1.66	1.92	2.19	1.04	0.407
Limimorpha	2.44	1.70	1.22	1.14	0.576

Table S4. Relative Abundances of the Top 20 Dominant Rumen Bacterial Genera in Lactating Bactrian Camels Fed Diets with Different Digestible Energy Levels

Taxa	CK	LE	ME	HE	*P*
Liminaster	1.82	1.33	1.30	1.27	0.610
Succinivibrio	0.42	0.59	0.48	3.66	0.481
Fibrobacter	0.98	0.78	1.12	1.69	0.211
CAG-269	0.97	1.05	0.78	0.93	0.148
UBA2804	1.04	0.58	1.03	0.73	0.814
Ruminococcus	0.42	1.25	0.73	0.73	0.336
UBA629	0.27	1.58	1.58	0.71	0.766
Minthosoma	0.87	0.67	0.78	0.59	0.650
Bovifimicola	0.52	0.34	0.86	1.07	0.229
Limivicinus	0.83	0.69	0.38	0.70	0.524
Ruminococcoides	0.49	0.71	0.54	0.77	0.596

Table S5. Genus-level P values after FDR correction

Genus	Raw *P* value	BH-adjusted *P* value
Brevundimonas	0.0680	0.989
Eubacterium_G	0.0220	0.989
Spyradosoma	0.0470	0.989
UBA3206	0.0431	0.989
UMGS1933	0.0424	0.989

Table S6. Detection of lignin-associated CAZymes in candidate MAGs

Enzyme type	Function	Gene count	Associated MAGs
Lignin-depolymerizing enzymes	Typical lignin-oxidizing enzymes, including AA1, AA2, and DyP-type peroxidases	0	Na
CE15	CE15 is mainly involved in cleaving linkages in lignin–carbohydrate complexes (LCCs)	7	MAG_153、MAG_348、MAG_386、MAG_6、MAG_698
AA4	AA4 participates in the oxidative transformation of lignin-derived aromatic compounds	3	MAG_203、MAG_440、MAG_757

Table S6. Detection of lignin-associated CAZymes in candidate MAGs

Enzyme type	Function	Gene count	Associated MAGs
GH28+CE15	Co-occurrence of pectin-degrading GH28 and LCC linkage-cleaving CE15 suggests potential synergistic deconstruction of plant cell-wall matrix components and LCCs.	1	MAG_900

The detected count represents the number of gene/enzyme entries assigned to the corresponding CAZy family across the 49 MAGs, rather than the number of MAGs containing that family.

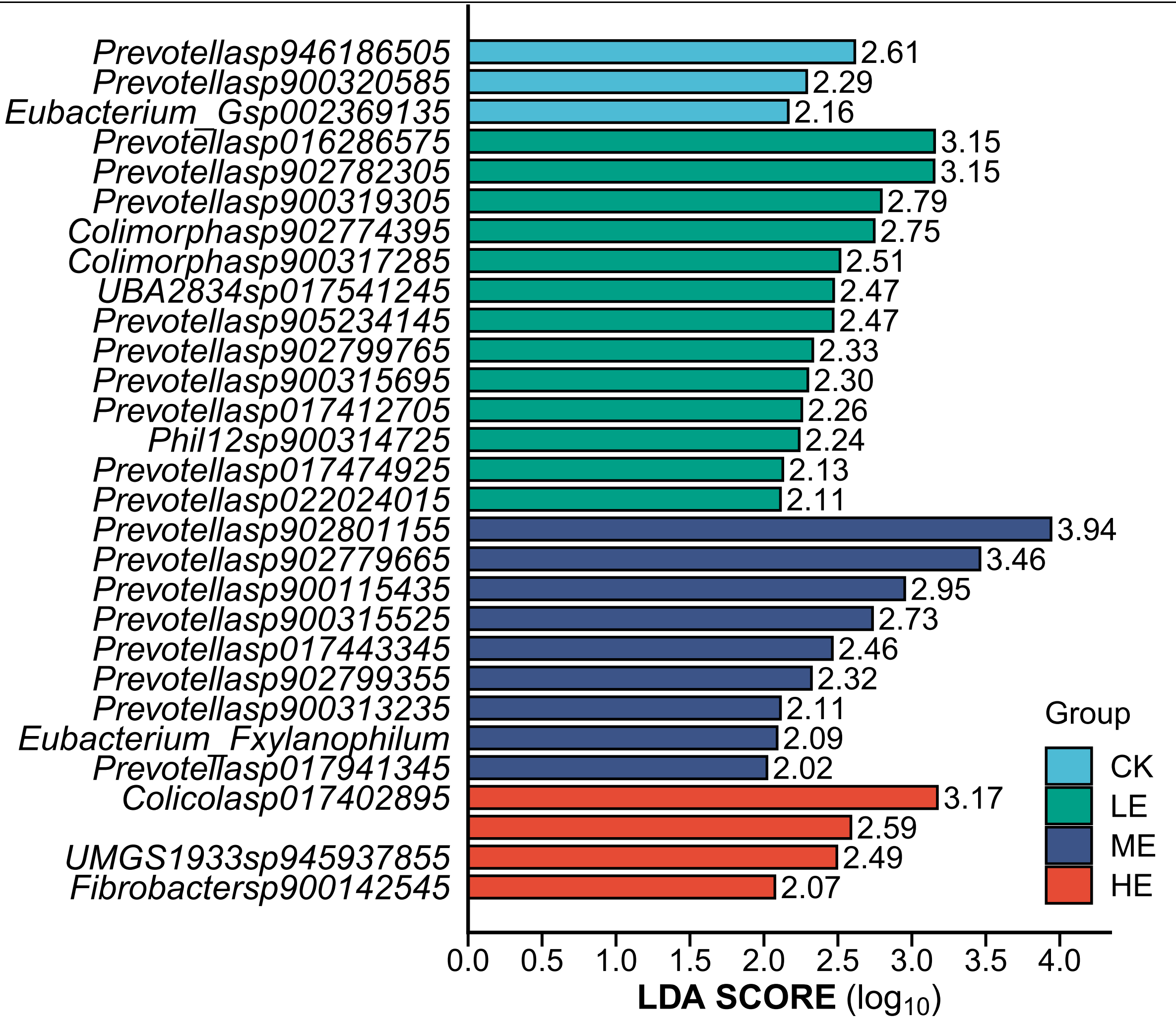

Figure S1. LEfSe analysis of differential rumen bacterial species in lactating Bactrian camels fed diets with different digestible energy levels.

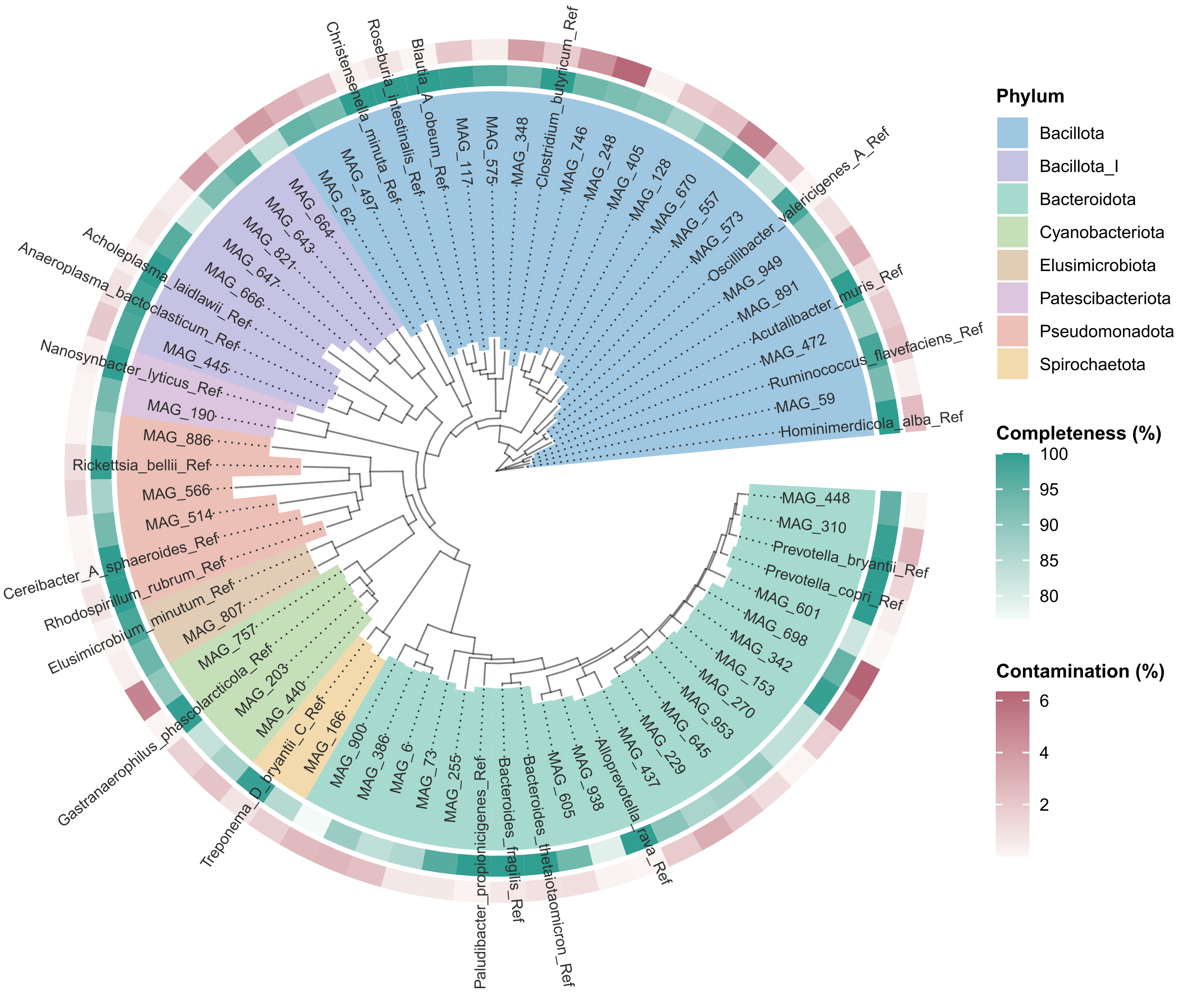

Figure S2. Circular phylogenetic tree of putatively novel MAGs from the camel rumen
